# Metabolic signature of cervical mucus in ewe breeds with divergent cervical sperm transport: a focus on metabolites involved in amino acid metabolism

**DOI:** 10.1007/s11306-023-02021-x

**Published:** 2023-06-20

**Authors:** Laura Abril-Parreño, Xavier Druart, Sean Fair, Anette Krogenaes

**Affiliations:** 1 Laboratory of Animal Reproduction, Department of Biological Sciences, School of Natural Sciences, Biomaterials Research Cluster, Bernal Institute, Faculty of Science and Engineering, University of Limerick, V94 T9PX Limerick, Ireland; 2 Station de Physiologie de la Reproduction et des Comportements, UMR 6175 INRA, CNRS-Université de Tours-Haras Nationaux, Institut National de la Recherche Agronomique, 37380 Nouzilly, France; 3 Faculty of Veterinary Medicine, Norwegian University of Life Sciences, 5003 1432 Ås, Norway

**Keywords:** Metabolome, Ovine, Cervix, Microbiota

## Abstract

**Introduction:**

Cervical artificial insemination (AI) with frozen-thawed semen in sheep has yielded unacceptably low pregnancy rates. The exception is in Norway where vaginal AI yields non-return rates in excess of 60%, which has been attributed to the ewe breed used.

**Objectives and methods:**

This study aimed to characterise, for the first time, the ovine follicular phase cervical mucus metabolome, with a focus on the amino acid profile. Cervical mucus was collected from four European ewe breeds with known differences in pregnancy rates following cervical AI with frozen-thawed semen. These were Suffolk (low fertility), Belclare (medium fertility), Norwegian White Sheep (NWS) and Fur (both high fertility).

**Results:**

A total of 689 metabolites were identified in the cervical mucus of all the four ewe breeds. Of these, 458 metabolites were altered by ewe breed, which had the greatest effect in the dataset (*P* < 0.05). We detected 194 metabolites involved in the amino acid pathway, of which 133, 56 and 63 were affected by ewe breed, type of cycle and their interaction, respectively (*P* < 0.05). N-methylhydantoin and N-carbamoylsarcosine (degradation products of creatinine pathway) exhibited the greatest fold change decrease in the Suffolk breed compared to Fur and NWS (*P* < 0.001). Oxidized metabolites were also decreased in Suffolk compared to high fertility breeds (*P* < 0.05). In contrast, other metabolites such as 3-indoxyl-sulfate, putrescine, cadaverine were significantly increased in Suffolk at the synchronised cycle.

**Conclusion:**

The suboptimal amino acid profile in the cervical mucus of the low fertility Suffolk breed may have negative consequences for sperm transport.

**Supplementary Information:**

The online version contains supplementary material available at 10.1007/s11306-023-02021-x.

## Introduction

In sheep, during non-surgical artificial insemination (AI) semen must be deposited either in the vagina or at the opening of the cervix as it is not possible to pass an inseminating pipette through the cervix due to the presence of cervical folds which are not concentrically aligned (Halbert, et al., 1990; Kershaw, et al., 2005). However, cervical AI with frozen-thawed semen has consistently yielded unacceptably pregnancy rates of less than 30% worldwide (See review by Fair et al. (2019). The only exception to this is in Norway, where pregnancy rates in excess of 60% are routinely achieved when farmers themselves vaginally (shot-in-the-dark) inseminate ewes with frozen-thawed semen to a natural oestrous (Paulenz, et al., 2005, 2007). The main reason for the success in Norway is the ewe breed used (Donovan, et al., 2004) and more specifically the ability of frozen-thawed sperm to traverse the cervix in some ewe breeds but not in others (Fair, et al., 2005).

Cervical mucus is a non-newtonian fluid composed of water (90–98%), inorganic ions, electrolytes, amino acids, lipids, carbohydrates and proteins (Tsiligianni, et al., 2001). Both the physical and biochemical properties of cervical mucus are modified by cyclic changes in hormone levels. In oestrous, in the lead up to ovulation, mucus production increases and mucus viscosity decreases (Gipson, 2001) allowing sperm with normal motility and morphology to traverse the cervix. In sheep, there are contradictory reports regarding the effect of progesterone and progestagens used for oestrous synchronisation on mucus production and composition. For example, Maddison et al. (2016) reported similar mucus volumes for a natural and a synchronised oestrous. In addition, progesterone has been associated with impaired sperm transport following synchronisation leading to reduced fertility (Lewis, et al., 2010; Maddison, et al., 2017).

Previous studies by our group were unable to find differences in oocyte quality (Fair, et al., 2006), hormonal profiles in the peri-ovulatory period (Fair, et al., 2007) or in the cervical anatomy and in gross mucus properties (volume and viscosity) that could explain the aforementioned ewe breed differences in pregnancy rates (Abril-Parreño, et al., 2021a). However, we did find ewe breed differences in *O*-glycan (Abril-Parreño, et al., 2021c) and sialic acid composition of cervical mucus (Abril-Parreño, et al., 2022b). These studies demonstrated that cervical mucus from the low fertility Suffolk breed had higher abundance of acidic glycans compared to Norwegian ewe breeds (Norwegian White Sheep (NWS) and Fur). Previously, Richardson et al. (2019) reported that sialic acid binding sites on frozen-thawed ram sperm could be blocked by incubating cervical mucus with free 6’-sialyllactose and sperm progressed further through cervical mucus *in vitro.*

In addition to the glycosylation of mucin proteins in the cervical mucus, other compounds such as metabolites secreted into mucus from the cervical epithelium or foreign organisms (e.g. bacteria) could also reflect a sub-optimal environment (Bokulich, et al., 2022). It has been recently reported that lipids (e.g. sphingolipids and long-chain unsaturated fatty acids) were strong predictors of genital inflammation, whereas predictions of vaginal microbiota and vaginal pH relied mostly on alterations in amino acid metabolism (Bokulich, et al., 2022). However, very little is known about the sheep cervical metabolome, how it can be modified by use of exogenous hormones used for oestrus synchronisation and its role in mediating sperm transport. Therefore, the objective of this study was to characterise, for the first time, the sheep cervical mucus metabolome, with a focus on the metabolites involved in the amino acid metabolism, of four European ewe breeds with known differences in pregnancy rates following cervical AI with frozen-thawed semen.

## Materials and methods

### Ethical approval

Protocols were developed in accordance with the *Cruelty to Animals Act* (Ireland 1876, as amended by European Communities regulations 2002 and 2005) and the European Community Directive 86/609/EC. In Ireland, the study was approved by the Teagasc Animal Ethics Committee (TAEC145/2017) and all animal procedures performed were conducted under experimental license from the Health Products Regulatory Authority. In Norway, the study was approved by the Norwegian Food Safety Authority (FOTS ID 13,168).

### Experimental design

This study was part of a larger study that aimed to interrogate the ewe breed differences in cervical mucus properties and composition across the oestrous cycle of both a synchronised (using progestogen sponges combined with equine chorionic gonadotropin) and a natural oestrous cycle. A full description of the experimental model and animal treatments have been previously described by Abril-Parreño et al. (2021a). For context a brief description of the experimental model is provided here. Cervical mucus samples were collected from four ewe breeds with known differences in pregnancy rates following cervical AI using frozen-thawed semen. These were Suffolk (low fertility; n = 29 ewes) and Belclare (medium fertility; n = 30 ewes) in Ireland as well as NWS and Fur (both high fertility) in Norway (n = 28 ewes in both breeds). Suffolk was the negative control due to the lowest pregnancy rates following cervical AI using frozen-thawed semen (Donovan, et al., 2004; Fair, et al., 2005). All ewes were maintained in-doors for the duration of the experiment with ad libitum access to forage and clean water.

At a natural cycle, all ewes were checked twice daily for signs of oestrus (observing standing oestrus in all ewes) over a 6 day period using a teaser ram with an apron fitted (no semen/seminal plasma was allowed to be deposited into the vagina of the ewe). At the synchronised cycle, ewes were synchronised using intravaginal progestagen vaginal sponges (20 mg Flugestone Acetate; Chronogest® vaginal sponges, Intervet, Boxmeer, The Netherlands), inserted on a random day of the cycle. After 14 days, the sponges were removed and ewes were administrated with equine chorionic gonadotropin (400 IU; Intervet, Boxmeer, The Netherlands) intramuscularly.

**Fig. 1:**
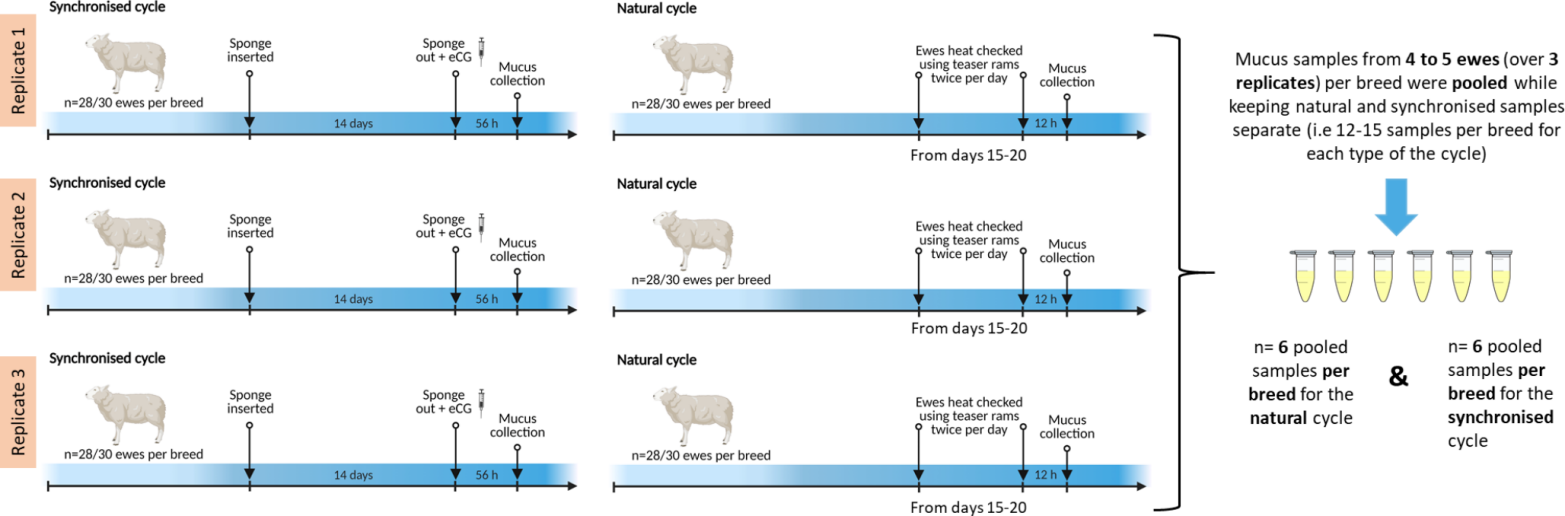
Experimental model showing the oestrous synchronisation design and sheep cervical mucus collection. Cervical mucus was collected from Suffolk, Belclare, Fur and Norwegian White Sheep ewes (n = 28 to 30 ewes per breed) at the follicular phase of a synchronised (14 day progestogen vaginal sponge + 400 IU equine chorionic gonadotropin at sponge removal) which was followed by a natural cycle (ewes were checked for signs of oestrous using teaser rams with aprons fitted twice per day during 6 days). Each cycle was repeated 3 times (using the same ewes) over a period of 6 months. After each mucus collection, samples were stored at 20 °C. Prior to the metabolomic analysis, samples were thawed on ice and pooled within breed and with type of the cycle (4–5 ewes over 3 replicates)

Cervical mucus was collected from each ewe at the follicular phase of the synchronised cycle at 56 h post sponge removal (replicated three times) and at a natural oestrus cycle 12 h post detection of standing oestrus, which was also replicated three times with the same ewes. All mucus collections were performed during the breeding season over a period of approximately six months from September to February (Fig. 1). The order in which these occurred was synchronised, natural, synchronised, natural, synchronised and natural. Synchronised ewes were not heat checked as is the norm for most fixed-time AI programmes, and cervical mucus was collected at a time (56 h post sponge removal) when ewes would normally be inseminated using frozen-thawed semen. Therefore, each ewe had 3 mucus samples collected at a natural and 3 mucus samples collected at a synchronised oestrus (synchronised cycle followed by a natural cycle, repeated 3 times). Briefly, to collect mucus, ewes were held in a standing position and the vulva was wiped clean with tissue soaked in disinfectant. Then, a duckbilled speculum (IMV Technologies, L’Aigle, France) with an internal light source was inserted into the vagina to locate the external cervical opening. Using an adapted suction pipette attached to a 20 mL syringe, all available mucus was suctioned from the cervical opening as well as from the fornix of the vagina. After each mucus collection, samples were transported (at room temperature) to the laboratory (maximum time = 1 h) for assessment of cervical mucus properties (weight, viscosity and colour; reported in Abril-Parreño et al. (2021a) following which the samples were stored at -20 °C until sample preparation for metabolomic analyses. Prior to metabolomic analysis all samples were thawed on ice once. Within breed, for each type of oestrous cycle (natural and synchronised), cervical mucus samples were pooled into 6 samples each containing 4 to 5 animals per breed (including three samples from the three collection times) and stored at -20 °C until processed. This yielded 6 pooled samples for each of the 4 ewe breeds at both a natural and synchronised oestrous cycle giving a total of 48 samples.

### Metabolomic analysis

Global metabolic profiles were determined from the samples by Metabolon, Inc (Morrisville, United States) as previously described by Evans et al. (2014). Briefly, cervical mucus samples were prepared using the automated MicroLab STAR system from Hamilton Company. Several recovery standards (Supplementary Table 1) were added at fixed concentration prior to the first step in the extraction process, to ensure injection and chromatographic consistency, and for alignment during data processing. Proteins were precipitated with methanol under vigorous shaking followed by centrifugation at 680 x *g* for 2 min (Geno/Grinder 2000, Glen Mills, New York, USA) to remove protein, dissociate small molecules bound to protein or trapped in the precipitated protein matrix, and to recover chemically diverse metabolites. The resulting extract was divided into five fractions. Two for analysis by two separate reverse phase (RP)/ultra-performance liquid chromatography (UPLC)-Mass spectrometry (MS)/MS methods with positive ion mode electrospray ionization (ESI). One for analysis by RP/UPLC-MS/MS with negative ion mode ESI. One for analysis by hydrophilic interaction chromatography (HILIC)/UPLC-MS/MS with negative ion mode ESI, and one sample was reserved for backup. Samples were placed on a TurboVap (Zymark) to remove the organic solvent (methanol). The sample extracts were stored overnight in nitrogen before preparation for analysis.

The details of Metabolon’s platform have been described previously (Ford, et al., 2020). The UPLC-MS/MS platform utilized was a Waters ACQUITY UPLC (Waters Corporation, Milford, MA) and a Thermo Scientific Q-Exactive high resolution/accurate mass spectrometer interfaced with a heated electrospray ionization (HESI-II) source and Orbitrap mass analyzer operated at 35,000 mass resolution. Sample extracts were dried, then reconstituted in solvents compatible to each of the methods mentioned above. Each reconstitution solvent contained a series of standards at fixed concentrations to ensure injection and chromatographic consistency. One aliquot was analysed using acidic positive ion conditions, chromatographically optimized for more hydrophilic compounds. In this method, the extract was gradient eluted from a C18 column (Waters UPLC BEH C18, 2.1 × 100 mm, 1.7 μm) using water and methanol, containing 0.05% perfluoropentanoic acid (PFPA) and 0.1% formic acid (FA). Another aliquot was also analysed using acidic positive ion conditions, however, it was chromatographically optimized for more hydrophobic compounds. In this method, the extract was gradient eluted from the same aforementioned C18 column using methanol, acetonitrile, water, 0.05% PFPA and 0.01% FA and was operated at an overall higher organic content. Another aliquot was analysed using basic negative ion optimized conditions using a separate dedicated C18 column. The basic extracts were gradient eluted from the column using methanol and water but with 6.5 mM ammonium bicarbonate at pH 8. The fourth aliquot was analysed via negative ionization following elution from a HILIC column (Waters UPLC BEH Amide, 2.1 × 150 mm, 1.7 μm) using a gradient consisting of water and acetonitrile with 10 mM ammonium formate (pH 10.8). The MS analysis alternated between MS and data-dependent MSn scans using dynamic exclusion. The scan range varied slighted between methods but covered 70 to 1000 *m/z*.

### Data extraction and compound identification

Raw data were extracted following which the peaks were identified and quality control processed using Metabolon’s hardware and software (DeHaven, et al., 2010). Compounds were identified by comparison to library entries of purified standards or recurrent unknown entities. Metabolon maintains a library based on authenticated standards that contains the retention time/index (RI), mass to charge ratio (m/z), and chromatographic data (including MS/MS spectral data) on all molecules present in the library. Furthermore, biochemical identifications are based on three criteria: retention index within a narrow RI window of the proposed identification, accurate mass match to the library +/- 10 ppm, and the MS/MS forward and reverse scores between the experimental data and authentic standards. The MS/MS scores are based on a comparison of the ions present in the experimental spectrum to the ions present in the library spectrum. While there may be similarities between these molecules based on one of these factors, the use of all three data points can be utilized to distinguish and differentiate biochemicals. The term “metabolite” was used for all the biochemicals (including amino acids) reported in this study.

### Biochemical quantification and statistical analyses

Peaks were quantified using area-under-the-curve. A data normalization step was performed to correct variations resulting from instrument inter-day tuning differences; median peak areas for each biochemical were registered as 1.00 prior to normalizing each data point proportionally. Following this, a global median was calculated for each sample in the dataset (across all metabolites in that sample) and the data were normalized by dividing by these values. As previously described by Do et al. (2018), biochemical data were logarithmically transformed, the missing values for a given metabolite were imputed with its observed minimum. Univariate and multivariate statistical analyses, including fold change, Welch’s two-sample t-test were conducted. A two-way ANOVA for each metabolite was used with breed and type of cycle as the main effects. From this model, both main effects and several ANOVA post hoc contrasts were generated (e.g., Natural Follicular Fur vs. Natural Follicular Belclare, etc.). For a given statistical test, we accounted for multiple testing using the false discovery rate (FDR) as determined by the q-value method of Storey and Tibshirani as described in Storey and Tibshirani (2003). Analysis by two-way ANOVA identified metabolites exhibiting significant interaction and main effects for experimental parameters of breed and type of cycle. *P*-values ≤ 0.05 were considered statistically significant and 0.10 ≤ *P*-values ≤ 0.05 were reported as trends. Principal component analysis (PCA), an unsupervised analysis that reduces the dimension of the data, was generated using Array Studio (OmicSoft). All samples described under ‘Metabolomic analyses’ (total n = 48) were included in the analyses. The comparisons done in this study were between (i) ewe breeds (ii) types of cycle and (iii) their interaction. As with all analyses when the interaction is significant it takes precedent and we have followed this rule.

### Pathway enrichment analysis

Pathway enrichment analysis allows the identification of small sets of pathway-associated metabolites from the large number of features present in a sample using a statistical enrichment-based approach. The pathway enrichment test uses the cumulative hypergeometric distribution to model the probability of observing ***k*** regulated items by chance, out of ***n*** total regulated items, in a category containing ***m*** items from a total population of ***N*** items. Pathway enrichment was calculated using the following formula: *(k/m)/(n-k)/(N-m)*, where *k*, was the number of significant metabolites in the pathway; *m*, the number of detected metabolites in the pathway; *n*, was the number of significant metabolites in both the study and pathway library; and, *N*, was the number of total metabolites in both the study and pathway library. The closer the probability (***p***) was to zero, the more unlikely by chance that the category was enriched for those items. .

## Results

### Metabolites in sheep cervical mucus

Levels of total protein (mg/ml) in cervical mucus were similar across all four ewe breeds at both types of oestrous cycle (*P* > 0.05). Cervical mucus collected at the natural cycle from Fur had a mean (± S.E.M) of 9.4 ± 5.40 mg/ml which was followed by Belclare (3.1 ± 0.56 mg/ml), Suffolk (1.9 ± 0.31 mg/ml) and NWS (1.8 ± 0.27 mg/ml). The highest protein content at the synchronised cycle was found in Belclare (3.2 ± 0.84 mg/ml) followed by Suffolk (3.1 ± 0.52), Fur (2.9 ± 0.67) and NWS (2.40 ± 0.29 mg/ml). Differences between both types of cycle were only found in Fur ewes, which had higher levels of protein at the natural cycle compared to the synchronised cycle (*P* < 0.05).

**Fig. 2:**
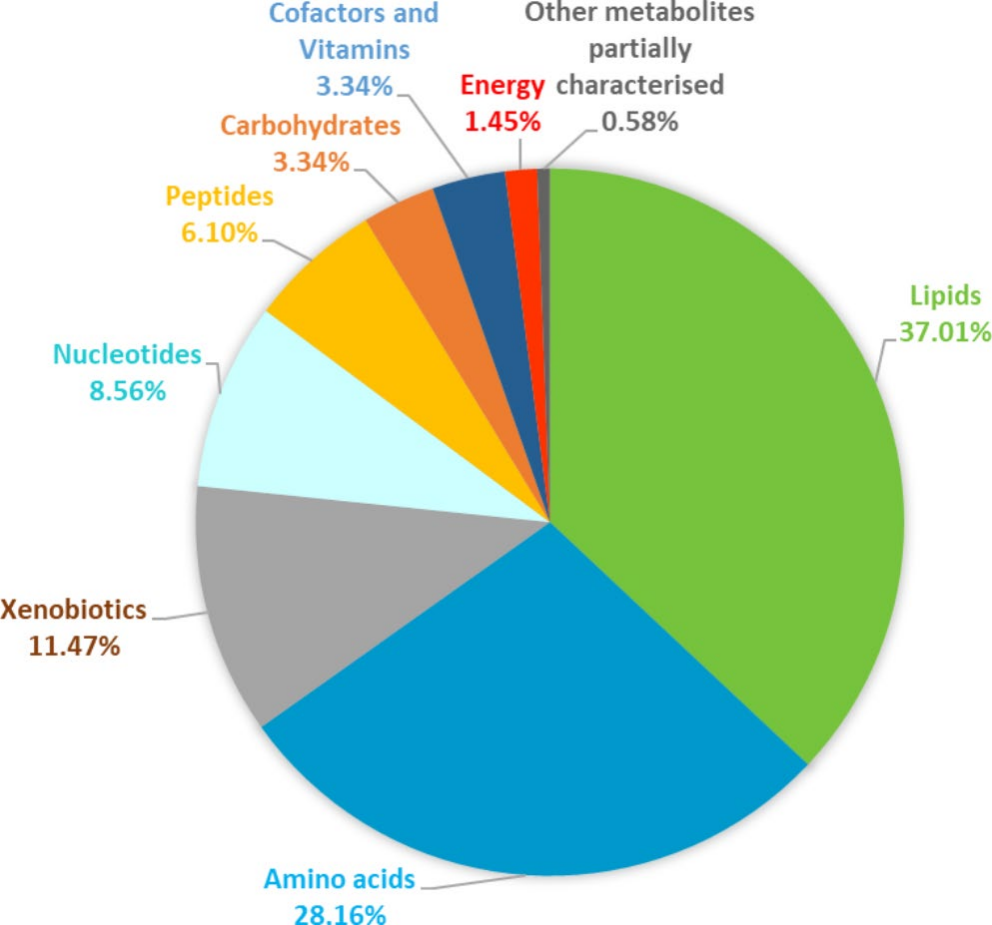
Pie chart showing the percentage of metabolites in each super pathway in sheep cervical mucus from all the four ewe breeds (Suffolk, Belclare, Fur and Norwegian White Sheep) at the follicular phase of both types of oestrous cycle

A total of 689 metabolites were consistently identified in the cervical mucus of all the four ewe breeds at both the synchronised and natural oestrous cycle. Of those identified metabolites, 37% were involved in lipid metabolism, 28% of molecules involved in amino acid metabolism, which were followed by xenobiotics, nucleotides, peptides, carbohydrates, cofactors and vitamins, energy and other metabolites which were partially characterised (Fig. 2).

**Fig. 3:**
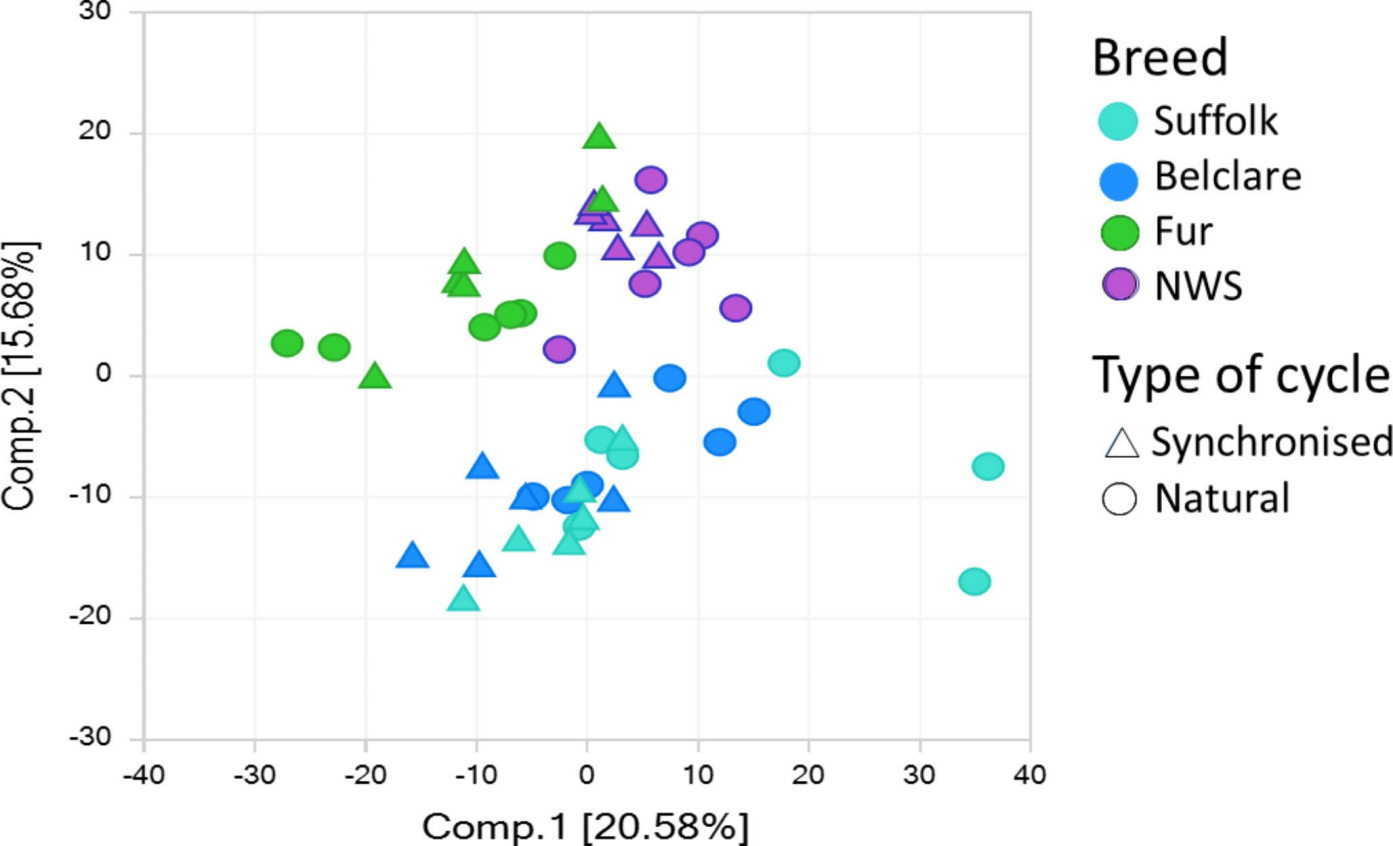
Principal component analysis (PCA) of total metabolites analysed in four ewe breeds (indicated with different colours) at both a natural and a synchronised cycle, represented as circles and triangles, respectively. Each dot represents a pooled sample. Significant separation based on the ewe breed suggesting that there were a number of significantly different metabolites between breeds that had high and low pregnancy rates. NWS = Norwegian White Sheep

Of those 689 metabolites, 458 were significantly altered by ewe breed, which had the strongest effect in the dataset (*P* ≤ 0.05) and 244 were affected by the type of oestrous cycle (*P* ≤ 0.05). There was a ewe breed by type of oestrous cycle interaction for 253 of the metabolites (*P* ≤ 0.05). In the PCA analysis there was a breed by type of the cycle interaction (*P* < 0.05) which was manifested by separation of the Suffolk with the NWS and Fur breeds at a synchronised but not at a natural cycle. (Fig. 3).

### Amino acid metabolites in sheep cervical mucus

A total of 194 metabolites involved in amino acid metabolism were consistently identified, spanning 15 subpathways in total from which 133 metabolites were affected by ewe breed, 56 by type of oestrous cycle and 63 metabolites were altered by the interaction of ewe breed and type of oestrous cycle (*P* ≤ 0.05).

**Fig. 4:**
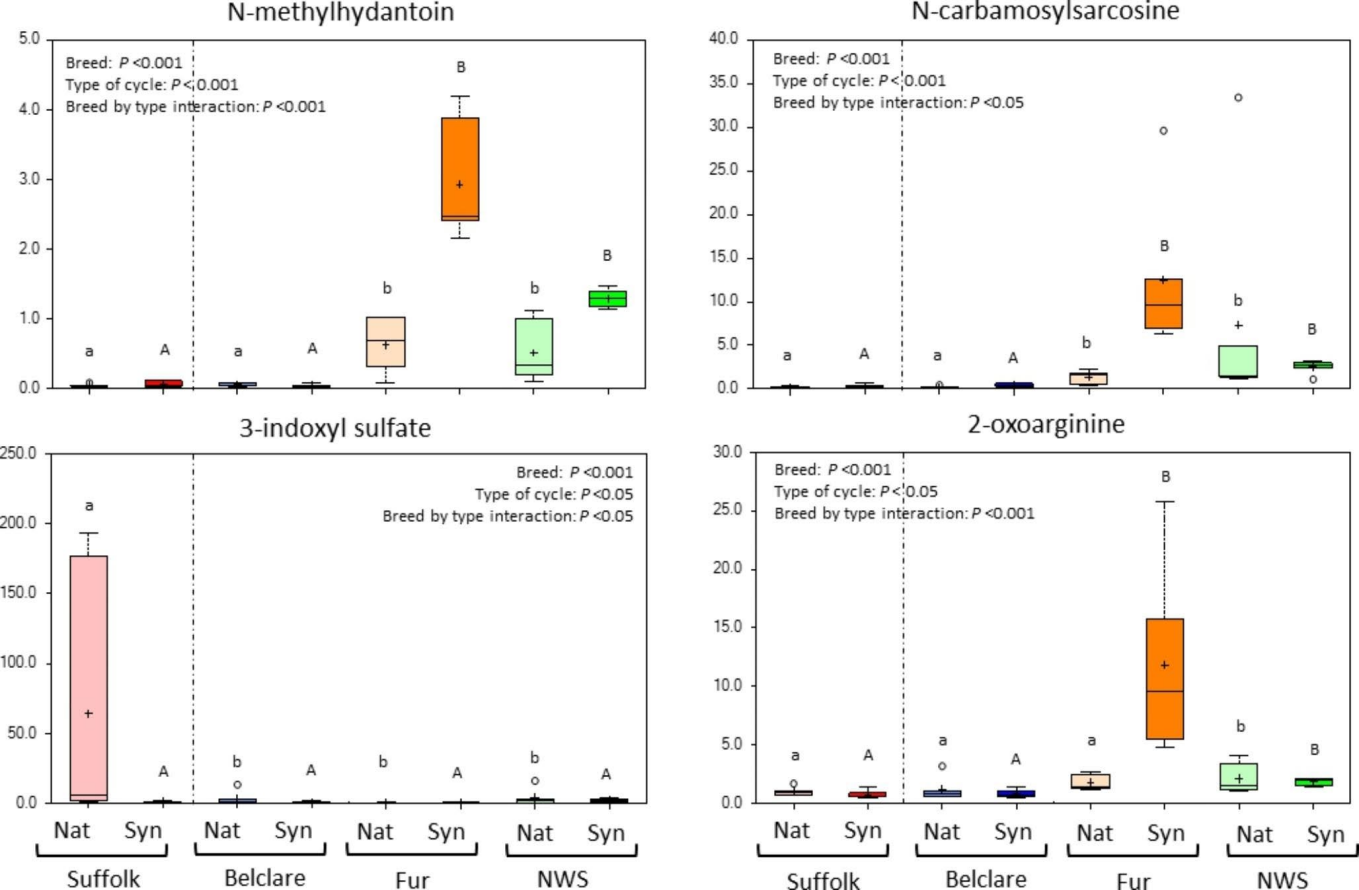
Box plots of N-methylhydantoin, N-carbamosylsarcosine, 3-indoxyl sulfate and 2-oxoarginine which all had a significant ewe breed by type of the cycle interaction. The central horizontal line represents the median value with outer boundaries depicting the upper and lower quartile limits. Error bars depict the minimum and maximum distributions, with + representing the mean value and ○ the extreme data point. Fold change differences are based on comparison of median values. Significant differences (*P* < 0.05) between the reference level (Suffolk) and the other ewe breeds at the natural cycle are denoted with different lower case superscripts (ab) while significant differences (*P* < 0.05) between them at the synchronised cycle are denoted with different upper case superscripts (AB). Abbreviations: Nat = natural cycle; Syn = synchronised cycle. NWS = Norwegian White Sheep

Of the 63 metabolites which had a ewe breed by type of the oestrous cycle interaction (*P* ≤ 0.05, Supplementary Tables 2 to 5), the top 4 most affected metabolites in amino acid metabolism with the highest fold changes are presented in Fig. 4. (*P* < 0.05). N*-*methylhydantoin, which is a product degradation of creatinine pathway, had the greatest fold change decrease (48.1) in Suffolk compared to Fur at the synchronised cycle (*P* < 0.001) but not at the natural cycle (Fig. 4). This was followed by *N*-carbamoylsarcosine which was decreased in Suffolk compared to NWS at the natural cycle as well as compared to Fur at the synchronised cycle. In contrast, 3-indoxyl sulfate was higher in the low fertility Suffolk breed compared to Belclare, NWS and Fur at the natural cycle (*P* < 0.001) but not at the synchronised (Fig. 4). 2-oxoarginine is also presented in Fig. 4 as it was the biochemical in the amino acid metabolism of the top 10 with decreased levels in both Norwegian ewe breeds at the synchronised cycle compared to Suffolk (*P* < 0.001).

**Fig. 5:**
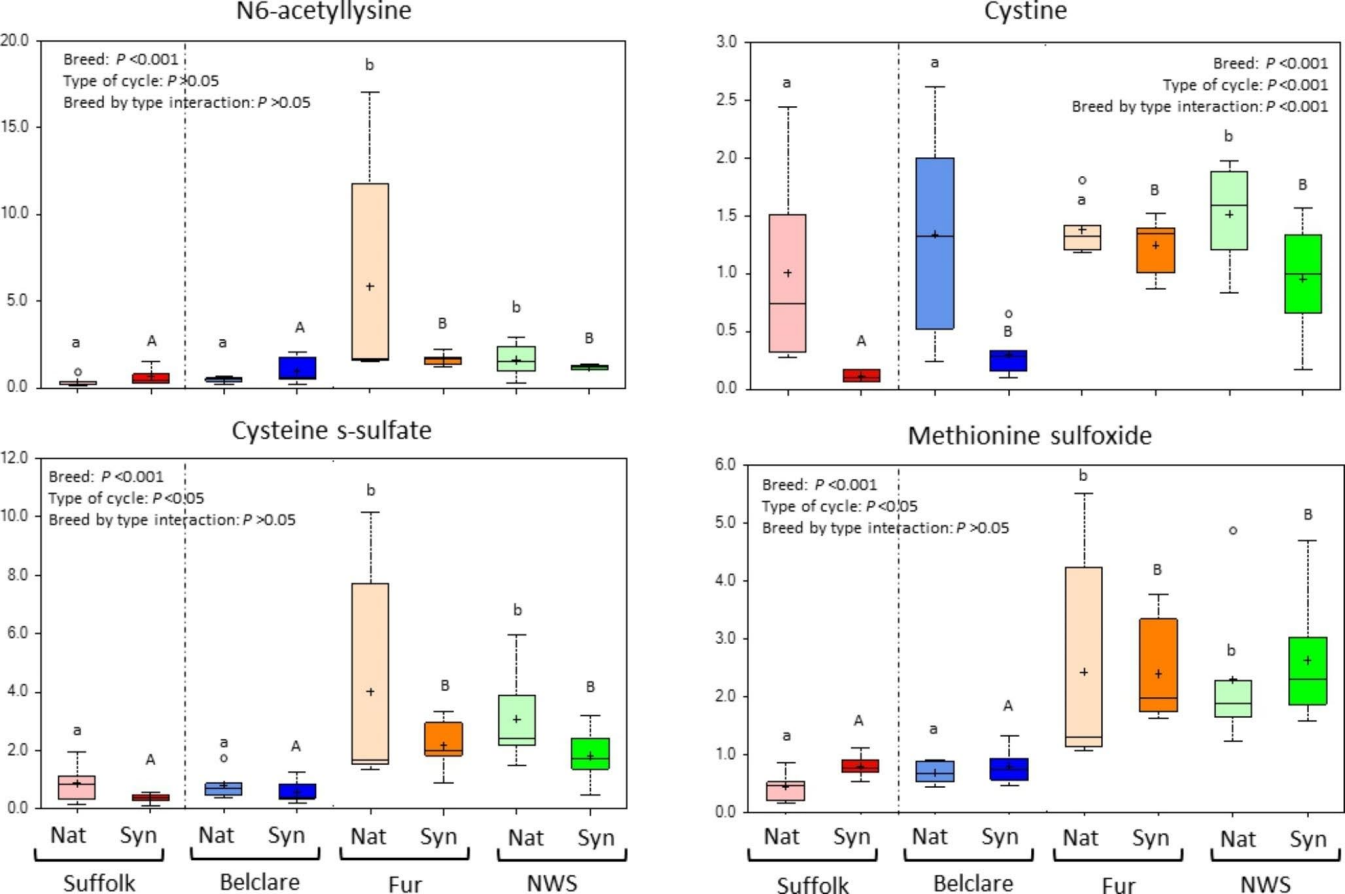
Box plots of N6-acetyllysine, cystine, cysteine s-sulfate and methionine sulfoxide which were all significantly affected by ewe breed. The central horizontal line represents the median value with outer boundaries depicting the upper and lower quartile limits. Error bars depict the minimum and maximum distributions, with + representing the mean value and ○ the extreme data point. Fold change differences are based on comparison of median values. Significant differences (*P* < 0.05) between the reference level (Suffolk) and the other ewe breeds at the natural cycle are denoted with different lower case superscripts (ab) while significant differences (*P* < 0.05) between them at the synchronised cycle are denoted with different upper case superscripts (AB). Abbreviations: Nat = natural cycle; Syn = synchronised cycle. NWS = Norwegian White Sheep

Regarding theWithin the differentially expressed metabolites affected by ewe breed (Supplementary Tables 2 to 5) N6-acetyllysine which is involved in the lysine metabolism was decreased in Suffolk compared to both Norwegian ewe breeds at both types of the cycle. This represented the highest fold change in the comparison of Suffolk against Fur at the natural cycle. This was followed by an 11.1-fold decrease of the cystine in Suffolk compared to Fur at the synchronised cycle (*P* < 0.001; Fig. 5). Furthermore, cysteine s-sulfate and methionine sulfoxide were decreased in Suffolk compared to NWS and Fur at both types of the oestrous cycle (*P* < 0.001; Fig. 5).

**Fig. 6:**
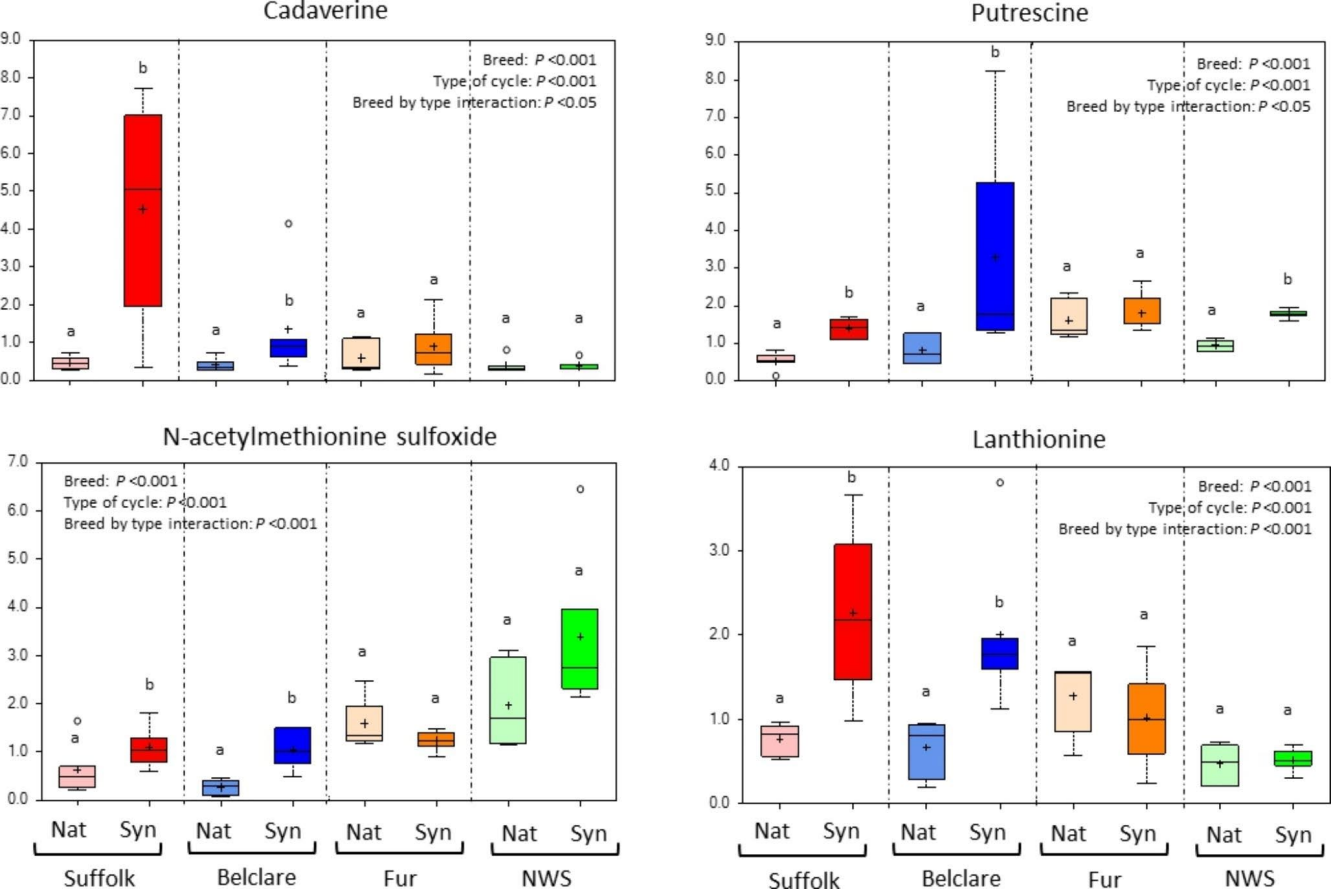
Box plots of the amino acids cadaverine, putrescine, N-acetylmethionine sulfoxide and lanthionine which were all significantly different between a natural and a synchronised oestrus. The central horizontal line represents the median value with outer boundaries depicting the upper and lower quartile limits. Error bars depict the minimum and maximum distributions, with + representing the mean value and ○ the extreme data point. Fold change differences are based on comparison of median values. ^ab^Different superscripts differ significantly between the natural and synchronised cycle within ewe breed (*P* < 0.05). Abbreviations: Nat = natural cycle; Syn = synchronised cycle. NWS = Norwegian White Sheep

There was 56 differentially expressed metabolites involved in amino acid metabolism significantly affected by type of the oestrous cycle (Synchronised versus Natural; Supplementary Tables 2 to 5). Within the metabolites with highest FC, cadaverine, involved in the lysine sub-pathway, was influenced the most by type of oestrous cycle in Suffolk, showing a decrease in the natural cycle compared to the synchronised cycle (*P* < 0.001; Fig. 6). Another metabolite (with a similar structure) is putrescine which had decreased levels in Belclare, Suffolk and NWS at the natural cycle compared to the synchronised cycle (*P* < 0.001; Fig. 6). The compounds *N-*acetylmethionine sulfoxide and lanthionine were decreased in Belclare and Suffok at the natural cycle compared to the synchronised (*P* < 0.001) but not in the Norwegian ewe breeds (Fig. 6).

Considering the greater metabolic sub-pathways affected by ewe breed and type of cycle interaction, the most enriched pathways and their pathway enrichment scores in brackets were creatine (2.2), guanidino and acetamido (1.36), histidine (1.26), glutamate (1.24), phenylalanine (1.21), tyrosine (1.14), glutathione (1.09) and tryptophan (1.09). Pathways enriched by ewe breed were creatine (1.51), guanidino and acetamido (1.51), tyrosine (1.26), glutathione (1.21), leucine, isoleucine and valine (1.15), lysine (1.11), glutamate (1.10) and histidine (1.04). However, the most enriched metabolic pathways in terms of type of oestrous cycle effect were tyrosine (1.42), guanidino and acetamido (1.41), glycine, serine and threonine (1.26), creatine (1.13), methionine, cysteine, SAM and taurine (1.06) and lysine (1.04).

## Discussion

This is the first published study that has characterised the metabolome of sheep cervical mucus. Using a unique model composed of ewe breeds with known differences in cervical sperm transport following cervical AI with frozen–thawed semen, we detected significant variations in the biochemical composition of the cervical mucus between high and low fertility ewe breeds and between a naturally occurring and hormonally synchronised oestrous cycles. Focusing on the amino acid profile, differences between ewe breeds and types of oestrous cycle were also identified. Interestingly, higher levels of compounds produced by mixed anaerobic bacteria such as 3-indoxyl sulfate, cadaverine and putrescine identified in the low fertility Suffolk breed could lead to new avenues of research on the cervical microbiome and its relationship with sperm transport across the cervix.

Creatine metabolism is essential to sustain ATP levels in tissues with high and fluctuating energy demand such as the female reproductive tract (Muccini, et al., 2021). For example, the creatine pathway is present in human (Castiglione Morelli, et al., 2020) and mouse follicular fluid, where it has been demonstrated that mouse follicular fluid creatine concentrations increase around the time of ovulation (Umehara, et al., 2018). There is also evidence that the creatine pathway is active in uterine tissue (Philip, et al., 2020) during phases of increased energy demand and in the myometrium during pregnancy (Charpigny, et al., 2003), thus suggesting that creatine metabolism in the myometrium is likely to be an important factor for the establishment and maintenance of pregnancy as well as contractility during labour. Although there are no previous studies about the effect of this on the cervical epithelium, our results show that the low fertility Suffolk breed had the lowest levels of two important metabolites (N-methylhydantoin and N-carbamoylsarcosine) produced during creatine metabolism. This is supported by our previous RNA-sequencing study, performed on cervical tissue of the same animals, where we reported a down-regulation of genes involved in smooth muscle contraction in the Suffolk breed (Abril-Parreño, et al., 2021b).

The 3-indoxyl sulfate is a tryptophan metabolism byproduct produced by mostly anaerobic bacteria (Wikoff, et al., 2009) which was found in higher levels in the cervical mucus of the low fertility Suffolk breed compared to the medium (Belclare) and high (Fur and NWS) fertility ewe breeds at the natural oestrous cycle. It has been reported that 3-indoxyl sulfate supports the survival of mixed microbial communities (Lee & Lee, 2010), increases inflammation and oxidative stress in macrophages (Adesso, et al., 2013) and in the intestinal mucosa (Vaziri, et al., 2013). This suggests a suboptimal cervical environment for sperm in the cervix of the low fertility Suffolk breed that could be produced by microbiota-derived metabolites. Highlighting that frozen-thawed sperm are impaired in getting across the Suffolk cervix, we propose that a high oxidative stress environment together with the deleterious effects of cryopreservation on sperm membrane (Peris-Frau, et al., 2020), could increase the susceptibility of frozen-thawed sperm to suffer oxidative damage and thus reduce sperm transport through the cervix.

There was a ewe breed effect on some mucus metabolites involved in the cysteine and methionine metabolism such as cystine, cysteine-s-sulfate and methionine sulfoxide, which were present in higher levels in the high fertility ewe breeds compared to Suffolk Sulfur-containing amino acids such as cysteine and methionine are prime targets of reactive oxygen species (Hoshi & Heinemann, 2001). The role of these oxidized residues have not been fully understood but it has been reported that the addition of cysteine and cystine during in vitro oocyte maturation decreased levels of oxidative stress (Zhou, et al., 2016). It has also been hypothesized that the oxidation of surface exposed methionines serves to protect other functionally essential residues from oxidative damage in proteins (Hoshi & Heinemann, 2001). Therefore, high levels of these oxidized compounds in high fertility ewe breeds could point to improved protection against a high oxidative environment.

Lanthionine and *N*-acetyl methionine sulfoxide, both which are involved in methionine metabolism, were significantly increased in the low fertility Suffolk breed at the synchronised cycle compared to the natural cycle. Some Gram-positive bacterial species produce lantibiotics, which are lanthionine containing antimicrobial peptides (Cotter, et al., 2005) that have been postulated to be induced by pathogens and cytokines as part of the innate host defense response (Diamond, et al., 2009), indicating an active immune response in the cervix of the Suffolk as we previously reported using RNA-sequencing analysis (Abril-Parreño, et al., 2022a).

Differences in mucus biochemical composition between the synchronised and natural cycle were also observed in two biogenic polyamines, cadaverine (produced by decarboxylation of lysine) and putrescine (a polyamine derived from ornithine and/or arginine), both commonly produced by mixed anaerobic vaginal bacteria resulting in a high risk of vaginal dysbiosis (Borgogna, et al., 2021). In our study, there were higher levels of cadaverine in Belclare (medium fertility) and the low fertility Suffolk at the synchronised cycle. Similarly, levels of putrescine in cervical mucus were also higher in the synchronised cycle compared to the natural in Suffolk, Belclare and NWS. Polyamines have been associated with activation of pro-inflammatory pathways (Al-Mushrif, et al., 2000; Mirmonsef, et al., 2012), which reduce the integrity of the epithelial barrier and consequently, increase the risk of infection (Srinivasan, et al., 2015).

Therefore, the increased levels of these products from bacterial metabolism could lead to an active immune response against bacteria as well as sperm compromised by cryopreservation. Although more studies are required to investigate this, in our previous RNA sequencing analysis of cervical tissue collected from the same animals (Abril-Parreño, et al., 2022a), we identified a heightened immune response in the cervix of the low fertility Suffolk breed. In addition, higher expression of a membrane transporter of polyamines (*SLC22A16*) and a polyamine oxidase (*PAOX*) was identified in Suffolk compared to Fur ewes (high fertility) at the follicular phase of a synchronised estrous cycle.

In conclusion, a large number of the differentially abundant metabolites were derived from mixed anaerobic bacteria in the Suffolk breed, indicating a suboptimal environment in the cervix which is likely to have knock-on negative consequences for sperm transport. In an increasing number of studies, the correlation between female infertility and the microbiota has been described (Campisciano, et al., 2017; Moreno & Simon, 2018; Wee, et al., 2018), both in the lower and/or in the upper female reproductive system. To better understand how the female reproductive tract microbiome mediates fertility, further work is required to identify the optimal cervical environment associated with positive sperm transport outcome.

## Electronic supplementary material

Below is the link to the electronic supplementary material.

Supplementary Material 1

## References

[CR1] Abril-Parreño L, Krogenæs AK, Byrne CJ, Donovan A, Stuen S, Caldas E, Diskin M, Druart X, Fair S (2021). Ewe breed differences in cervical anatomy and cervicovaginal mucus properties: An international study. Theriogenology.

[CR3] Abril-Parreño L, Meade KG, Krogenæs AK, Druart X, Fair S, Cormican P (2021). Conserved and breed-specific differences in the cervical transcriptome of sheep with divergent fertility at the follicular phase of a natural oestrus cycle. Bmc Genomics.

[CR5] Abril-Parreño, L., Wilkinson, H., Krogenæs, A., Morgan, J., Gallagher, M. E., Reid, C., Druart, X., Fair, S., & Saldova, R. (2021c). Identification and characterization of O-linked glycans in cervical mucus as biomarkers of sperm transport: A novel sheep model. *Glycobiology*. 10.1093/glycob/cwab085 PMC8881736 34379775

[CR2] Abril-Parreño L, Meade KG, Krogenæs AK, Druart X, Cormican P, Fair S (2022). Ewe breed differences in the cervical transcriptome at the follicular phase of a synchronised oestrous cycle. Bmc Genomics.

[CR4] Abril-Parreño, L., Morgan, J., Krogenæs, A., Druart, X., Cormican, P., Gallagher, M. E., Reid, C., Meade, K., Saldova, R., & Fair, S. (2022b). Biochemical and molecular characterisation of sialylated cervical mucins in sheep. *Biol Reprod*. 10.1093/biolre/ioac077 PMC9382375 35470857

[CR6] Adesso S, Popolo A, Bianco G, Sorrentino R, Pinto A, Autore G, Marzocco S (2013). The Uremic Toxin Indoxyl Sulphate enhances macrophage response to LPS. PLOS ONE.

[CR7] Al-Mushrif S, Eley A, Jones BM (2000). Inhibition of chemotaxis by organic acids from anaerobes may prevent a purulent response in bacterial vaginosis. Journal Of Medical Microbiology.

[CR9] Bokulich NA, Łaniewski P, Adamov A, Chase DM, Caporaso JG, Herbst-Kralovetz MM (2022). Multi-omics data integration reveals metabolome as the top predictor of the cervicovaginal microenvironment. PLOS Computational Biology.

[CR10] Borgogna, J. C., Shardell, M. D., Grace, S. G., Santori, E. K., Americus, B., Li, Z., Ulanov, A., Forney, L., Nelson, T. M., Brotman, R. M., Ravel, J., & Yeoman, C. J. (2021). Biogenic amines increase the Odds of bacterial vaginosis and affect the growth of and lactic acid production by vaginal Lactobacillus spp. *Applied And Environment Microbiology* 87. 10.1128/AEM.03068-20 PMC8117770 33674429

[CR11] Campisciano G, Florian F, D’Eustacchio A, Stanković D, Ricci G, De Seta F, Comar M (2017). Subclinical alteration of the cervical-vaginal microbiome in women with idiopathic infertility. Journal Of Cellular Physiology.

[CR12] Castiglione Morelli MA, Iuliano A, Schettini SCA, Petruzzi D, Ferri A, Colucci P, Viggiani L, Ostuni A (2020). Metabolic changes in follicular fluids of patients treated with recombinant versus urinary human chorionic gonadotropin for triggering ovulation in assisted reproductive technologies: A metabolomics pilot study. Archives Of Gynecology And Obstetrics.

[CR13] Charpigny G, Leroy MJ, Breuiller-Fouché M, Tanfin Z, Mhaouty-Kodja S, Robin P, Leiber D, Cohen-Tannoudji J, Cabrol D, Barberis C, Germain G (2003). A functional genomic study to identify differential gene expression in the preterm and term human myometrium. Biology Of Reproduction.

[CR14] Cotter PD, Hill C, Ross RP (2005). Bacterial lantibiotics: Strategies to improve therapeutic potential. Current Protein And Peptide Science.

[CR15] DeHaven CD, Evans AM, Dai H, Lawton KA (2010). Organization of GC/MS and LC/MS metabolomics data into chemical libraries. Journal of Cheminformatics.

[CR16] Diamond G, Beckloff N, Weinberg A, Kisich KO (2009). The roles of antimicrobial peptides in innate host defense. Current pharmaceutical design.

[CR17] Do KT, Wahl S, Raffler J, Molnos S, Laimighofer M, Adamski J, Suhre K, Strauch K, Peters A, Gieger C, Langenberg C, Stewart ID, Theis FJ, Grallert H, Kastenmüller G, Krumsiek J (2018). Characterization of missing values in untargeted MS-based metabolomics data and evaluation of missing data handling strategies. Metabolomics: Official journal of the Metabolomic Society.

[CR19] Donovan A, Hanrahan JP, Kummen E, Duffy P, Boland MP (2004). Fertility in the ewe following cervical insemination with fresh or frozen-thawed semen at a natural or synchronised oestrus. Animal Reproduction Science.

[CR20] Evans, A. M., Br, B., Liu, Q., Mitchell, M. W., Rj, R., Dai, H., Sj, S., DeHaven, C. D., & Lad, M. (2014). High Resolution Mass Spectrometry Improves Data Quantity and Quality as Compared to Unit Mass Resolution Mass Spectrometry in High- Throughput Profiling Metabolomics. *Metabolomics* 4, 1–3.

[CR22] Fair S, Hanrahan JP, O’Meara CM, Duffy P, Rizos D, Wade M, Donovan A, Boland MP, Lonergan P, Evans AC (2005). Differences between Belclare and Suffolk ewes in fertilization rate, embryo quality and accessory sperm number after cervical or laparoscopic artificial insemination. Theriogenology.

[CR23] Fair S, Hanrahan JP, Ward F, O’Meara CM, Duffy P, Donovan A, Lonergan P, Evans AC (2006). The difference in embryo quality between Belclare and Suffolk ewes is not due to differences in oocyte quality. Theriogenology.

[CR21] Fair S, Hanrahan JP, Donovan A, Duffy P, O’Meara CM, Lonergan P, Evans AC (2007). Hormonal relationships during the periovulatory period among ewe breeds known to differ in fertility after cervical artificial insemination with frozen thawed semen. Animal Reproduction Science.

[CR24] Fair S, Meade KG, Reynaud K, Druart X, de Graaf SP (2019). The biological mechanisms regulating sperm selection by the ovine cervix. Reproduction.

[CR25] Ford L, Kennedy AD, Goodman KD, Pappan KL, Evans AM, Miller LAD, Wulff JE, Wiggs BR, Lennon JJ, Elsea S, Toal DR (2020). Precision of a clinical Metabolomics profiling platform for Use in the identification of inborn errors of metabolism. J Appl Lab Med.

[CR26] Gipson IK (2001). Mucins of the human endocervix. Frontiers In Bioscience : A Journal And Virtual Library.

[CR27] Halbert, G. W., Dobson, H., Walton, J. S., & Buckrell, B. C. (1990). The structure of the cervical canal of the ewe. *Theriogenology* 33, 977 – 92. 10.1016/0093-691x(90)90060-7 16726794

[CR28] Hoshi T, Heinemann S (2001). Regulation of cell function by methionine oxidation and reduction. The Journal of physiology.

[CR29] Kershaw CM, Khalid M, McGowan MR, Ingram K, Leethongdee S, Wax G, Scaramuzzi RJ (2005). The anatomy of the sheep cervix and its influence on the transcervical passage of an inseminating pipette into the uterine lumen. Theriogenology.

[CR30] Lee JH, Lee J (2010). Indole as an intercellular signal in microbial communities. Fems Microbiology Reviews.

[CR31] Lewis RA, Taylor D, Natavio MF, Melamed A, Felix J, Mishell D (2010). Effects of the levonorgestrel-releasing intrauterine system on cervical mucus quality and sperm penetrability. Contraception.

[CR33] Maddison JW, Rickard JP, Mooney E, Bernecic NC, Soleilhavoup C, Tsikis G, Druart X, Leahy T, de Graaf SP (2016). Oestrus synchronisation and superovulation alter the production and biochemical constituents of ovine cervicovaginal mucus. Animal Reproduction Science.

[CR32] Maddison JW, Rickard JP, Bernecic NC, Tsikis G, Soleilhavoup C, Labas V, Combes-Soia L, Harichaux G, Druart X, Leahy T, de Graaf SP (2017). Oestrus synchronisation and superovulation alter the cervicovaginal mucus proteome of the ewe. J Proteomics.

[CR34] Mirmonsef P, Zariffard MR, Gilbert D, Makinde H, Landay AL, Spear GT (2012). Short-chain fatty acids induce pro-inflammatory cytokine production alone and in combination with toll-like receptor ligands. American Journal Of Reproductive Immunology.

[CR35] Moreno I, Simon C (2018). Relevance of assessing the uterine microbiota in infertility. Fertility And Sterility.

[CR36] Muccini, A. M., Tran, N. T., de Guingand, D. L., Philip, M., Della Gatta, P. A., Galinsky, R., Sherman, L. S., Kelleher, M. A., Palmer, K. R., Berry, M. J., Walker, D. W., Snow, R. J., & Ellery, S. J. (2021). Creatine Metabolism in Female Reproduction, Pregnancy and Newborn Health. *Nutrients* 13. 10.3390/nu13020490 PMC7912953 33540766

[CR38] Paulenz H, Söderquist L, Adnøy T, Nordstoga AB, Berg A (2005). Effect of vaginal and cervical deposition of semen on the fertility of sheep inseminated with frozen-thawed semen. The Veterinary Record.

[CR37] Paulenz H, Adnøy T, Söderquist L (2007). Comparison of fertility results after vaginal insemination using different thawing procedures and packages for frozen ram semen. Acta veterinaria Scandinavica.

[CR39] Peris-Frau, P., Soler, A. J., Iniesta-Cuerda, M., Martín-Maestro, A., Sánchez-Ajofrín, I., Medina-Chávez, D. A., Fernández-Santos, M. R., García-Álvarez, O., Maroto-Morales, A., Montoro, V., & Garde, J. J. (2020). Sperm cryodamage in ruminants: Understanding the Molecular Changes Induced by the Cryopreservation process to optimize sperm quality. *International Journal Of Molecular Sciences* 21. 10.3390/ijms21082781 PMC7215299 32316334

[CR40] Philip M, Snow RJ, Gatta PAD, Bellofiore N, Ellery SJ (2020). Creatine metabolism in the uterus: Potential implications for reproductive biology. Amino Acids.

[CR42] Richardson L, Hanrahan JP, Tharmalingam T, Carrington SD, Lonergan P, Evans ACO, Fair S (2019). Cervical mucus sialic acid content determines the ability of frozen-thawed ram sperm to migrate through the cervix. Reproduction.

[CR44] Srinivasan, S., Morgan, M. T., Fiedler, T. L., Djukovic, D., Hoffman, N. G., Raftery, D., Marrazzo, J. M., & Fredricks, D. N. (2015). Metabolic signatures of bacterial vaginosis. *mBio* 6, e00204-15. 10.1128/mBio.00204-15 PMC4453549 25873373

[CR45] Storey JD, Tibshirani R (2003). Statistical significance for genomewide studies. Proc Natl Acad Sci U S A.

[CR46] Tsiligianni T, Karagiannidis A, Brikas P, Saratsis P (2001). Physical properties of bovine cervical mucus during normal and induced (progesterone and/or PGF2α) estrus. Theriogenology.

[CR47] Umehara T, Kawai T, Goto M, Richards JS, Shimada M (2018). Creatine enhances the duration of sperm capacitation: A novel factor for improving in vitro fertilization with small numbers of sperm. Human reproduction (Oxford England).

[CR48] Vaziri ND, Yuan J, Norris K (2013). Role of urea in intestinal barrier dysfunction and disruption of epithelial tight junction in chronic kidney disease. American Journal Of Nephrology.

[CR49] Wee BA, Thomas M, Sweeney EL, Frentiu FD, Samios M, Ravel J, Gajer P, Myers G, Timms P, Allan JA, Huston WM (2018). A retrospective pilot study to determine whether the reproductive tract microbiota differs between women with a history of infertility and fertile women. Australian And New Zealand Journal Of Obstetrics And Gynaecology.

[CR50] Wikoff WR, Anfora AT, Liu J, Schultz PG, Lesley SA, Peters EC, Siuzdak G (2009). Metabolomics analysis reveals large effects of gut microflora on mammalian blood metabolites. Proc Natl Acad Sci U S A.

[CR51] Zhou Z, Jia RX, Zhang G, Wan Y, Zhang Y, Fan Y, Wang Z, Huang P, Wang F (2016). Using cysteine/cystine to overcome oxidative stress in goat oocytes and embryos cultured in vitro. Molecular Medicine Reports.

